# Are the PHQ-9 and GAD-7 Suitable for Use in India? A Psychometric Analysis

**DOI:** 10.3389/fpsyg.2021.676398

**Published:** 2021-05-13

**Authors:** Jeroen De Man, Pilvikki Absetz, Thirunavukkarasu Sathish, Allissa Desloge, Tilahun Haregu, Brian Oldenburg, Leslie C. M. Johnson, Kavumpurathu Raman Thankappan, Emily D. Williams

**Affiliations:** ^1^Department of Family Medicine and Population Health, University of Antwerp, Antwerp, Belgium; ^2^Collaborative Care Systems Finland, Tampere University, Tampere, Finland; ^3^University of Eastern Finland, Kuopio, Finland; ^4^Population Health Research Institute, McMaster University, Hamilton, ON, Canada; ^5^Melbourne School of Population and Global Health, University of Melbourne, Melbourne, VIC, Australia; ^6^Department of Family and Preventive Medicine, School of Medicine, Emory University, Atlanta, GA, United States; ^7^Hubert Department of Global Health, Rollins School of Public Health, Emory University, Atlanta, GA, United States; ^8^Department of Public Health and Community Medicine, Central University of Kerala, Kasaragod, India; ^9^School of Health Sciences, University of Surrey, Guildford, United Kingdom

**Keywords:** Patient Health Questionnaire, India, measurement invariance, depression, generalized anxiety disorder assessment, sum score reliability, confirmatory bifactor modeling, omega hierarchical

## Abstract

**Background:**

Cross-cultural evidence on the factorial structure and invariance of the PHQ-9 and the GAD-7 is lacking for South Asia. Recommendations on the use of unit-weighted scores of these scales (the sum of items’ scores) are not well-founded. This study aims to address these contextual and methodological gaps using data from a rural Indian population.

**Methods:**

The study surveyed 1,209 participants of the Kerala Diabetes Prevention Program aged 30–60 years (*n* at risk of diabetes = 1,007 and *n* with diabetes = 202). 1,007 participants were surveyed over 2 years using the PHQ-9 and the GAD-7. Bifactor-(S – 1) modeling and multigroup confirmatory factor analysis were used.

**Results:**

Factor analysis supported the existence of a somatic and cognitive/affective subcomponent for both scales, but less explicitly for the GAD-7. Hierarchical omega values were 0.72 for the PHQ-9 and 0.76 for the GAD-7. Both scales showed full scalar invariance and full or partial residual invariance across age, gender, education, status of diabetes and over time. Effect sizes between categories measured by unit-weighted scores versus latent means followed a similar trend but were systematically higher for the latent means. For both disorders, female gender and lower education were associated with higher symptom severity scores, which corresponds with regional and global trends.

**Conclusions:**

For both scales, psychometric properties were comparable to studies in western settings. Distinct clinical profiles (somatic-cognitive) were supported for depression, and to a lesser extent for anxiety. Unit-weighted scores of the full scales should be used with caution, while scoring subscales is not recommended. The stability of these scales supports their use and allows for meaningful comparison across tested subgroups.

**Clinical Trial Registration:**

Australia and New Zealand Clinical Trials Registry: ACTRN12611000262909

http://www.anzctr.org.au/Trial/Registration/TrialReview.aspx?id=336603&isReview=true.

## Introduction

Depressive and anxiety disorders are among the ten leading causes of global disability (Vos et al., 2015) and more than 80% of people who have mental disorders reside in low- and middle income countries (LMICs) (World Health Organization, 2008). In India, depressive and anxiety disorders have been shown to both have a crude prevalence of 3.3% and were responsible for 33.8% (29.5–38.5) and 19.0% (15.9–22.4) of disease-adjusted life years attributable to mental disorders (Sagar et al., 2020).

Self-reported measurement tools are crucial to estimate the burden of depressive and anxiety disorders at population level, to determine how this burden relates to subgroup characteristics (e.g., sociodemographic characteristics, other health conditions, etc.), and to measure the effect of public health interventions. At individual level, these tools can enhance the reliability of diagnoses and their ease of use makes them particularly useful in settings with poor mental health service provision and a lack of specialized staff (Mughal et al., 2020). However, most of the established self-reported measurement tools have been developed and evaluated in Europe and North America and may not perform in an equivalent way in different cultures or settings (Dere et al., 2015; Mughal et al., 2020).

The Patient Health Questionnaire (PHQ-9) and Generalized Anxiety Disorder (GAD-7) assessment can be used as screening tools as well as measures of symptom severity for depression (PHQ-9) and different types of anxiety (GAD-7) (Kroenke et al., 2001; Spitzer et al., 2006). Both tools are based on the Diagnostic and Statistical Manual of Mental Disorders criteria and have been found to be valid measures for detecting and monitoring depression or anxiety disorders in western countries (Kroenke et al., 2010). In South Asia, a region home to one quarter of the world’s population, assessment of these scales‘ essential psychometric properties is lacking (Lamela et al., 2020). Below, we will discuss why assessment of such properties is crucial and their current level of evidence, focusing on: (1) the factorial structure; (2) the use of unit-weighted scores (i.e., the sum score of the item responses); and (3) invariance across subgroups.

The factorial structure of data from a specific population can provide empirical support for the potential existence of subdimensions of depression and anxiety disorders which can differ across cultures and settings (Leong and Tak, 2003). For instance, studies have revealed that somatic symptoms are more common in Indian people with depression compared with western populations (Grover et al., 2010). For the PHQ-9 and the GAD-7, which were initially intended to be used as unidimensional scales, a variety of measurement models have been proposed based on confirmatory factor analysis (CFA) investigations (Doi et al., 2018a,b; Moreno et al., 2019; Lamela et al., 2020). In these studies, mainly conducted in western settings, researchers have found both scales to fit unidimensional and multidimensional models (Lamela et al., 2020), a second-order model specifically for the GAD-7 (Doi et al., 2018a) and a bifactor model specifically for the PHQ-9 (Doi et al., 2018b). A recurrent finding is the identification of a factor consisting of items reflecting a somatic aspect and a factor consisting of items reflecting a cognitive/affective aspect (Beard and Björgvinsson, 2014; Lamela et al., 2020). For both disorders, these subdimensions correspond with clinical representations and pathophysiologic insights suggesting different subtypes of these conditions (Portman et al., 2011; Duivis et al., 2013; Lamela et al., 2020). For depression in particular, distinguishing between these subtypes has been shown important with regards to treatment and prognosis (Lamela et al., 2020). However, to our knowledge, no studies have assessed the factorial structure of these scales in India or anywhere else in South Asia. To examine the cross-cultural validity of these subdimensions, evidence on the factorial structure of these scales based on data from different settings is needed.

A key question when using self-reported measurement tools is to what extent the unit-weighted scores (i.e., the sum score of all or a subset of item responses) can be interpreted as a unidimensional representation of a specific construct. For instance, to what extent does the sum of all scale item scores represent depressive or anxiety symptom severity as an overarching construct. Items of these scales may belong to different subdimensions which may preclude the interpretation of the sum of their scores. In addition, clinicians or researchers may want to sum item responses belonging to one of these subdimensions and use this score as a reflection of that specific subdimension. Recent studies have defended scoring the total scale as well as its subdimensions for the PHQ-9 (Doi et al., 2018b; Lamela et al., 2020) and for the GAD-7 (Doi et al., 2018a) guided by the goodness of fit of CFA models. These recommendations are problematic for several reasons. First, Reise et al. (2013) argue that model selection based on CFA rarely informs researchers on the degree of multidimensionality such that it may justify the use of unit-weighted scores of subscales or total scales. Second, methodologists have called into question if specifying both, total and subdimension scores of the same scale can have an added value (Rodriguez et al., 2016a). Third, consensus is lacking on the choice of the most adequate CFA model for both, the PHQ-9 and GAD-7. To guide researchers on this matter, alternative analytic techniques have been proposed such as the Haberman (2008) procedure (Haberman, 2008) and the use of model-based reliability indices (Rodriguez et al., 2016b). To our knowledge, these techniques have not been applied yet to either the PHQ-9 or the GAD-7. Application of these techniques to data collected from different settings may redress current inconsistencies in recommendations on the use of unit-weighted scores.

Measurement invariance of a scale is an essential psychometric property when studying scale scores over time or between subgroups of a population (Vandenberg and Lance, 2000). Measurement invariance across subgroups corresponds to a latent construct being represented by the scale items in a similar way and suggests that this construct has a similar meaning to these groups. This implies that assessing invariance is crucial for the comparison of subgroups. When measurement invariance is violated across subgroups, prevalence or severity of a disorder may be under- or overestimated across these groups. Finally, analysis of invariance can provide insight into how the interpretation of a scale and the perception of an illness may differ across subgroups. This may have consequences with regards to the diagnosis and how people cope with their illness. Lack of invariance can lead to substantial bias when comparing different subgroups, especially when using unit-weighted scores (Steinmetz, 2013). Moreover, in addition to scalar invariance which is required to compare subgroups through a structural equation modeling (SEM) framework, the use of unit-weighted scores requires invariant indicator reliability (Vandenberg and Lance, 2000). Invariance of the PHQ-9 and GAD-7 scales has been supported by studies conducted in western settings for gender, ethnic and sociodemographic differences, but only few studies assessed invariance for age and over time (Hinz et al., 2017; Lamela et al., 2020). Moreover, other depression measures have shown non-invariance across different age groups (Estabrook et al., 2015). Only a handful of studies have tested invariance in LMICs and were focused on a specific population such as college students, pregnant women, etc. (Miranda and Scoppetta, 2018). To our knowledge, no studies have assessed any form of invariance of the PHQ-9 and GAD-7 in South Asian populations.

In sum, essential psychometric properties of the PHQ-9 and the GAD-7 remain underexplored among LMIC populations, in particular in South Asia. Furthermore, recommendations on the use of unit-weighted scores of these scales have been poorly supported.

The aim of this study was to simultaneously address these contextual and methodological gaps based on a state-of-the-art analytic approach and using data from a rural Indian population. Specifically, we aimed to: (1) assess existence of subdimensions in this population by assessing the factorial structure of data collected through these scales; (2) determine how precisely total and subscale scores reflect their intended constructs; (3) assess invariance across subgroups of age, gender, level of education, status of diabetes (at risk of versus with type 2 diabetes [T2D]) and different measurement occasions; and (4) if invariance could be established, compare the results of unit-weighted scores with latent means analysis in the assessment of differences in symptom severity across the same subgroups. With this last objective, we sought to assess: (1) the accuracy of unit-weighted scores when using latent mean levels as a standard; and (2) whether differences across subgroups correspond with regional trends that would support the validity of our data.

## Materials and Methods

### Participants

The analysis was based on data collected from participants who took part in the baseline, cross-sectional, community-based survey of a diabetes prevention program in the state of Kerala in India: the Kerala Diabetes Prevention Program (K-DPP). A detailed description of the study design, participant screening and recruitment has been previously published (Sathish et al., 2013, 2017, 2019). Briefly, K-DPP was a cluster randomized controlled trial conducted in 60 randomly selected polling areas (electoral divisions) from a taluk (sub-district) in Trivandrum district of Kerala state. People aged 30–60 years were selected randomly from the electoral roll of the 60 polling areas and were approached at their households by trained data collectors. We screened 3,421 individuals for eligibility and those with a history of diabetes or other major chronic conditions, taking drugs influencing glucose tolerance (e.g., steroids), and who were illiterate in the local language were excluded (*n* = 835). Potentially eligible individuals (*n* = 2586) were screened with the Indian Diabetes Risk Score, and those with a score of ≥60 (*n* = 1529) were invited to undergo a 2-h oral glucose tolerance test (OGTT) at community-based clinics. Of these, 1,209 attended the clinics, of which 1,007 individuals were at high risk for developing diabetes and 202 were diagnosed with diabetes. Participant screening and recruitment were completed between January and October 2013. The 1,007 individuals at high risk were followed-up after 1 and 2 years of enrollment. These follow-up points were used for the analysis of invariance at different measurement occasions. Mean age of participants was 46.0 (*SD*: 7.5), 45.8% were female, and 95% were married. 25.3, 51.3, and 23.4% attended primary, secondary and higher education, respectively (Sathish et al., 2017).

### Measures and Data Collection

Both the nine-item PHQ-9 and the seven-item GAD-7 use 4-point Likert-scaled items ranging from 0 (not at all) to 3 (nearly every day) (Kroenke et al., 2001; Spitzer et al., 2006). For the GAD-7, items 4, 5, and 6 have been found to reflect a somatic dimension (Rutter and Brown, 2017). For the PHQ-9, this was the case for items 3, 4, and 5 and in some studies items 7 and 8 (Lamela et al., 2020). The scales were translated to Malayalam and back-translated to English and were pilot tested. Interviews were administered by trained interviewers.

### Data-Analysis

#### Confirmatory Factor Analysis

To evaluate whether our data supported a two dimensional model, a selection of models was tested with specifications based on theory and findings from previous studies. The aim of this analysis was to test if a model with one or two factors would be acceptable, rather than to select a specific model solely based on a better model fit. For the PHQ-9, we tested a correlated 2-factor model with items 3–4–5 loading onto one factor as was proposed by others (Lamela et al., 2020). For the GAD-7, we tested a 1-factor model with and without correlated residuals for items 4, 5, and 6 and a correlated 2-factor model with items 4, 5, and 6 loading onto one factor (Beard and Björgvinsson, 2014; Rutter and Brown, 2017; Doi et al., 2018a). Assessment of these models was based on their χ^2^-values, the item indicators’ loadings and the following sample-corrected for non-normal data goodness of fit indices (Brosseau-Liard et al., 2012) with target values as proposed by Hu and Bentler (1999): the comparative fit index (CFI) (≥0.95), the Tucker-Lewis index (TLI) (≥0.95), the root mean square error of approximation (RMSEA) (≤0.06), and the standardized root mean square residual (SRMR) (≤0.08). Since items’ distributions departed from normality, we used maximum likelihood estimation with robust (Huber–White) standard errors and a scaled test statistic that is (asymptotically) equal to the Yuan–Bentler test statistic.

#### Haberman Procedure

This procedure assesses whether the subscore provides a more accurate estimate of the construct it measures than the total score (Haberman, 2008). The proportional reduction of mean squared error (PRMSE) based on total scores was compared with the PRMSE based on subscale scores. In case the latter would be smaller, there is no psychometric justification to report the subscale scores. In addition, a hypothesis test was performed justifying the reporting of subscale scores if Olkin’s *Z* statistic was higher than 1.64 (Sinharay, 2019). However, this procedure does not test whether subscale scores provide meaningful information, while taking into account the total score (Reise et al., 2013). To assess this, we used the indices described in the next paragraph.

#### Model-Based Psychometric Indices

Rodriguez et al. (2016a, b) proposed the following indices to assess the degree to which total and subscale scores reflect their intended constructs. Total omega (omegaT) estimates the proportion of variance in the unit-weighted total score due to all common factors including the general and group factors (Zinbarg et al., 2005). Hierarchical omega (omegaH) estimates the proportion variance due to a general factor. Omega hierarchical subscale (omegaHS) estimates the variance due to a specific group factor while controlling for the general factor. Recommended minimum values were described for OmegaH (0.70) and for OmegaHS (0.50) (Reise et al., 2013; Rodriguez et al., 2016a).

The indices were calculated based on a confirmatory bifactor modeling approach (Reise et al., 2013) using the semTools package in R (Jorgensen et al., 2020). A bifactor model was deemed appropriate to calculate these indices as it is a less restrictive model (than, e.g., a hierarchical model) and as the structure of the response data was assumed to be consistent with a bifactor structure: i.e., a single general trait reflecting the target construct and the presence of subdomain constructs due to clusters of similar items (Rodriguez et al., 2016a). Bifactor models can be specified by all items loading onto a general factor as well as on group factors representing the subdomains. In addition, group factors are assumed to be uncorrelated with other group factors as well as with the general factor. However, since only two group factors were present, these bifactor models will be unidentified, which implies that an infinite number of hierarchical factor models can be found for the same covariance matrix (Zinbarg et al., 2007). To address this problem, we used a modified version of the traditional bifactor model: the bifactor-(*S* − 1) model described by Eid et al. (2016). The name of this model refers to the number of specific group factors being less than the actual number of the scale’s subdimensions being considered. This modification makes the discarded group factor a reference group for the general factor and solves the identification problem (Eid et al., 2016).

#### Invariance

Invariant indicator reliabilities of unit-weighted scores across groups requires residual or strict invariance which was tested using multigroup confirmatory factor analysis (MGCFA) (Vandenberg and Lance, 2000). The following levels of invariance were assessed: equal form (i.e., configural invariance), equality of factor loadings (i.e., metric invariance), equality of indicator intercepts (scalar invariance), and equality of residuals (i.e., residual invariance) (Vandenberg and Lance, 2000). However, for residual invariance to be analogous to indicator reliability invariance, the last step requires invariance of factor variances which was tested first (Vandenberg and Lance, 2000). Criteria of invariance between nested models included a difference in CFI < −0.01 combined with difference in RMSEA < 0.015 or a non-significant scaled χ-square difference test (Putnick and Bornstein, 2016).

#### Group Differences

For the unit-weighted scores, standardized effect sizes between different subgroups were calculated using robust regression of the unit-weighted scores based on MM-estimation (i.e., an extension of the maximum likelihood estimate method). Standardized effect sizes of unit-weighted scores were compared with the standardized effect sizes of the difference in latent mean levels estimated through multigroup structural equation modeling. Data were analyzed using R software with the packages “lavaan” (Rosseel, 2012) and “semTools” (Jorgensen et al., 2020).

#### Missing Data

Missing data occurred in 0.4% of the GAD-7 data, in 3.4% of the PHQ-9 data and in 0.0% of the demographic variables (sex, education, and age). It was deemed implausible that the probability of missing data would significantly differ in specific groups or cases, assuming they were missing completely at random (MCAR). This was supported by Little’s test hypothesis not being rejected for a subset of the GAD-7 and demographic variables (*p* = 0.30) and the PHQ-9 and demographic variables (*p* = 0.07). For these reasons, complete case analysis was preferred.

### Ethical Approval

The study was approved by the Institutional Ethics Committee of the Sree Chitra Tirunal Institute for Medical Sciences and Technology, Trivandrum, Kerala, and by the Human Research Ethics Committees of Monash University, Australia and the University of Melbourne, Australia. The study was also approved by the Health Ministry Screening Committee of the Government of India.

## Results

### Factor Structure

**FIGURE 1:**
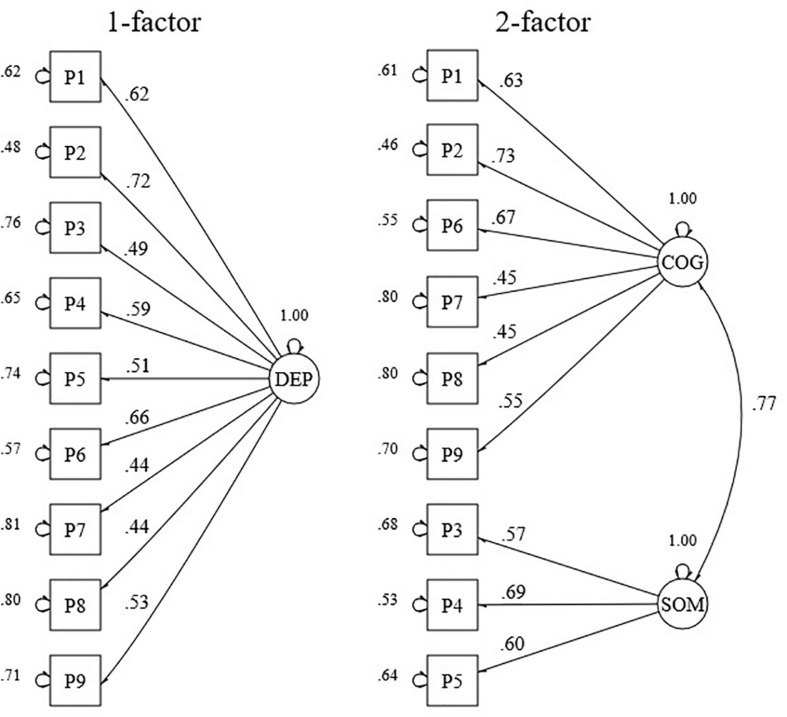
Standardized factor loadings and error variances for a 1- and a 2-factor model of the PHQ-9. DEP, depression; COG, cognitive; SOM, somatic. (*n* = 1207).

**FIGURE 2:**
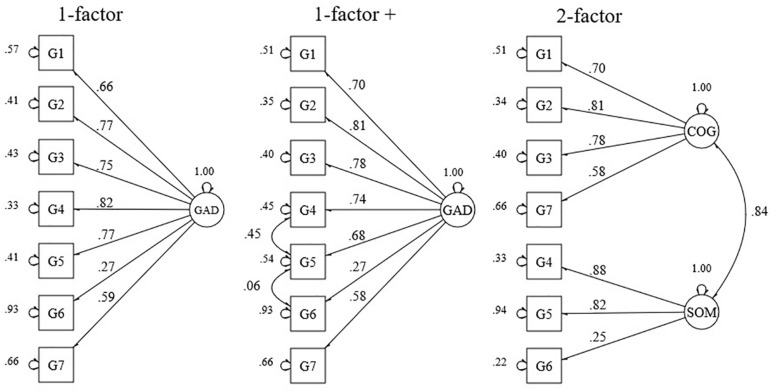
Standardized factor loadings and error variances for a 1- and a 2-factor model, and a model with correlated residuals of the GAD-7. GAD, generalized anxiety disorder; COG, cognitive; SOM, somatic. (*n* = 1205).

As mentioned previously, the aim of this analysis was to assess the existence of a somatic and a cognitive subdimension in the response data. For this purpose, we assessed whether model fit criteria of 1- and 2-factor models were acceptable (see Figures 1, 2 and Table 1). CFA of the models proposed for the PHQ-9 revealed an acceptable fit for a 2-factor model, but not for a 1-factor model (see Table 1). The factors of the 2-factor model were highly correlated (*r* = 0.77). Factor loading estimates revealed that the indicators were strongly related to their purported factors (range λ = 0.45–0.73) with *p*-values below 0.001 (see Figure 1).

**TABLE 1:** Model fit of the PHQ-9 (*n* = 1207) and the GAD-7 (*n* = 1205).

**Scale**	**Model**	**χ^2^**	**df**	***p*-value**	**CFI**	**TLI**	**RMSEA**	**90% CI**	**SRMR**
PHQ-9	1-factor	148.37	27	<0.001	0.907	0.876	0.083	0.070–0.096	0.050
	2-factor	96.34	26	<0.001	0.947	0.926	0.064	0.051–0.078	0.038
GAD-7	1-factor	133.13	14	<0.001	0.935	0.903	0.114	0.097–0.132	0.039
	1-factor +	33.97	14	0.001	0.988	0.979	0.053	0.033–0.075	0.024
	2-factor	46.61	21	<0.001	0.982	0.971	0.062	0.044–0.082	0.032

*df, degrees of freedom; CFI, comparative fit index; TLI, Tucker–Lewis index; RMSEA, root mean square error of approximation; 90% CI, 90% confidence interval for RMSEA; SRMR, standardized root mean square residual*.

CFA of the models proposed for the GAD-7 revealed an acceptable fit for a 1-factor model with correlated residuals between items 3, 5, and 6, and a 2-factor model (see Table 1). The correlation between the cognitive and somatic factor of the 2-factor model was high (*r* = 0.84). Factor loading estimates revealed that the indicators were strongly related to their purported factors (range λ = 0.58–0.88), except for item 6 (λ = 0.27) (see Figure 2). For all parameters, *p*-values were below 0.001 except for the correlation between the residuals of items 5 and 6 (*p*-value = 0.05).

### Haberman Procedure

PRMSE based on subscale scores was smaller than the PRMSE of the total score for the somatic dimension of the PHQ-9 (0.57 vs. 0.63) and the GAD-7 (0.63 vs. 0.81) and for the cognitive dimension of the GAD-7 (0.80 vs. 0.83). This was confirmed by a formal hypothesis test with the Olkin’s *Z* statistic lower than 1.64. These results imply that there is no added value in reporting these subscale scores as total scores would be a relatively more precise indicator of subscale true scores than the actual subscale scores. For the cognitive dimension of the PHQ-9, the PRMSE based on the subscale scores was higher than the PRMSE based on the total score (0.69 vs. 0.62), which indicates that this subscale score is a more precise indicator of its true score.

### Model-Based Psychometric Indices

**FIGURE 3:**
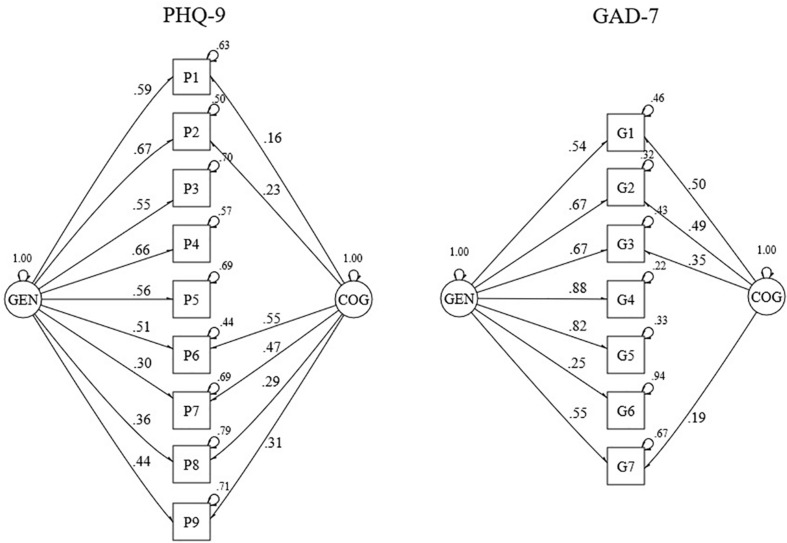
Standardized factor loadings and error variances for a bifactor-(*S* – 1) model of the PHQ-9 (*n* = 1207) and the GAD-7 (*n* = 1205). GEN, general factor; COG, cognitive group factor.

As mentioned earlier, a bifactor-(*S* − 1) model was fitted to calculate these indices (see Figure 3). For both scales, the somatic factor was discarded making it the reference group for the general factor. Model fit of these models was acceptable for the PHQ-9 and excellent for the GAD-7 (see Table 2). For both scales, estimates of omegaH above the recommended minimum value and the difference between omegaT and omegaH was relatively small (i.e., 0.08 for both scales), which indicates that the general factor is the major determinant of the variance underlying the unit-weighted total scale scores. Estimates of the omegaHS were small for both scales, reflecting little unique variance due to any specific group factor.

**TABLE 2:** Bifactor-(*S* − 1) model fit and reliability indices of the PHQ-9 (*n* = 1207) and the GAD-7 (*n* = 1205).

	**PHQ-9**	**GAD-7**
**Indices of fit**		
χ^2^	133.530	27.987
df	21	21
*p*-value	<0.001	<0.001
CFI	0.948	0.991
TLI	0.910	0.982
RMSEA	0.071	0.049
90% CI	0.059–0.082	0.028–0.071
SRMR	0.033	0.026
**Reliability indices**		
OmegaT	0.80	0.84
OmegaH	0.72	0.76
OmegaHS somatic	0.27	0.27
OmegaHS cognitive	0.22	0.23

*df, degrees of freedom; CFI, comparative fit index; TLI, Tucker–Lewis index; RMSEA, root mean square error of approximation; 90% CI, 90% confidence interval for RMSEA; SRMR, standardized root mean square residual. OmegaT, total omega; omegaH, hierarchical omega; and omegaHS, omega hierarchical subscale*.

### Invariance

#### PHQ-9

Applying CFA to the different subgroups of gender, age, education, T2D status, and different measurement occasions, resulted in good model fit, except for the age group 30–40 and the group with higher education (see Table 3). Scalar invariance could be established across all different subgroups (see Table 4). Invariance of latent factor variance could be established for all subgroups, except for education. Full or partial residual invariance could be established across all different subgroups. Partial residual invariance across all three measurement occasions required free estimation of residuals of at least six items. Per two measurement occasions, partial invariance could be obtained by freeing two (T0–T1) to five residuals (T1–T2).

**TABLE 3:** Assessment of dimensional invariance of the PHQ-9 and GAD-7 across demographic subgroups, status of diabetes and over time.

		**PHQ-9**								**GAD-7**							
		***N***	**χ ^2^**	**df**	***p***	**CFI**	**TLI**	**RMSEA**	**SRMR**	***N***	**χ ^2^**	**df**	***p***	**CFI**	**TLI**	**RMSEA**	**SRMR**
Gender	Female	553	65.737	26	0.000	0.940	0.917	0.067	0.044	554	33.022	12	0.001	0.978	0.962	0.073	0.035
	Male	654	57.140	26	0.000	0.949	0.929	0.059	0.041	651	14.268	12	0.284	0.997	0.995	0.024	0.022
Age	30–40	326	66.215	26	0.000	0.878	0.831	0.089	0.060	324	17.777	12	0.123	0.988	0.979	0.049	0.032
	41–50	493	49.860	26	0.003	0.956	0.939	0.059	0.043	495	26.334	12	0.010	0.984	0.973	0.062	0.033
	51–60	388	61.913	26	0.000	0.940	0.917	0.072	0.046	386	19.646	12	0.074	0.987	0.977	0.057	0.026
Education	Primary	305	57.584	26	0.000	0.930	0.903	0.083	0.052	305	25.109	12	0.014	0.979	0.964	0.076	0.029
	Secondary	619	56.576	26	0.000	0.952	0.934	0.058	0.042	617	15.282	12	0.226	0.996	0.993	0.028	0.022
	Higher	283	39.294	26	0.046	0.930	0.904	0.049	0.052	283	26.647	12	0.009	0.966	0.940	0.086	0.047
T2D-Status	At risk	1006	85.341	26	0.000	0.947	0.926	0.065	0.040	1003	25.562	12	0.012	0.991	0.984	0.046	0.023
	T2D	201	40.088	26	0.038	0.944	0.922	0.061	0.051	202	14.962	12	0.244	0.989	0.981	0.050	0.039
Time	T0	1006	85.341	26	0.000	0.947	0.926	0.065	0.040	1003	25.562	12	0.012	0.991	0.984	0.046	0.023
	T1	982	39.152	26	0.047	0.984	0.978	0.033	0.031	981	21.062	12	0.049	0.993	0.988	0.039	0.022
	T2	963	52.996	26	0.001	0.974	0.965	0.046	0.038	963	36.948	12	0.000	0.978	0.961	0.067	0.029

*df, degrees of freedom; CFI, comparative fit index; TLI, Tucker–Lewis index; RMSEA, root-mean-square error of approximation; SRMR, standardized root mean square residual: T2D, type two diabetes*.

**TABLE 4:** Assessment of measurement invariance of the PHQ-9 and GAD-7 across demographic subgroups, over time and status of diabetes.

**PHQ-9**		**Items constrained**	**χ ^2^**	**df**	***p***	**CFI**	**RMSEA**	**SRMR**	**Δχ ^2^**	***p***	**Δ CFI**	**Δ RMSEA**	**Δ SRMR**
Gender	Configural		122.124	52	<0.001	0.945	0.063	0.039	−	−	−	−	−
	Metric		121.398	59	<0.001	0.948	0.057	0.043	3.666	0.817	0.004	–0.006	0.004
	Scalar		131.714	66	<0.001	0.948	0.054	0.044	8.101	0.324	–0.001	–0.003	0.001
	Factor variance		137.501	68	<0.001	0.944	0.056	0.069	5.047	0.080	–0.004	0.001	0.025
	Residual*		191.250	75	<0.001	0.894	0.072	0.069	39.371	<0.001	–0.054	0.018	0.025
	Partial residual*	2,6,7	142.568	72	<0.001	0.941	0.055	0.052	11.250	0.081	–0.007	0.001	0.007
Age	Configural		176.513	78	<0.001	0.932	0.072	0.044	−	−	−	−	−
	Metric		177.109	92	<0.001	0.935	0.065	0.055	114.809	0.648	0.003	–0.007	0.011
	Scalar		198.213	106	<0.001	0.934	0.061	0.057	172.130	0.245	–0.001	–0.004	0.002
	Factor var		209.552	110	<0.001	0.927	0.063	0.084	98.314	0.043	–0.007	0.002	0.027
	Residual*		208.579	124	<0.001	0.928	0.059	0.069	21.647	0.248	–0.006	–0.002	0.012
Education	Configural		156.348	78	<0.001	0.941	0.064	0.043	−	−	−	−	−
	Metric		186.644	92	<0.001	0.926	0.066	0.063	29.826	0.008	–0.016	0.002	0.020
	Partial metric	7	167.316	90	<0.001	0.940	0.060	0.052	13.392	0.341	–0.001	–0.004	0.009
	Scalar		185.049	104	<0.001	0.941	0.055	0.053	12.461	0.569	0.001	–0.004	0.001
	Factor variance		218.204	108	<0.001	0.916	0.065	0.110	19.521	<0.001	–0.025	0.009	0.057
	Residual*		274.559	122	<0.001	0.859	0.079	0.109	58.147	<0.001	–0.082	0.023	0.056
	Partial residual*	6,7	200.944	118	<0.001	0.932	0.056	0.064	20.026	0.129	–0.009	0.000	0.012
T2D status	Configural		131.678	52	<0.001	0.946	0.064	0.038	−	−	−	−	−
	Metric		130.505	59	<0.001	0.948	0.060	0.044	58.489	0.558	0.001	–0.005	0.006
	Scalar		139.259	66	<0.001	0.949	0.056	0.044	43.797	0.735	0.001	–0.004	0.000
	Factor variance		136.175	68	<0.001	0.951	0.054	0.049	0.8415	0.657	0.002	–0.002	0.005
	Residual*		144.056	75	<0.001	0.945	0.054	0.050	11.655	0.233	–0.004	–0.001	0.006
Time	Configural		173.821	78	<0.001	0.968	0.050	0.033	−	−	−	−	−
	Metric		197.636	92	<0.001	0.963	0.049	0.046	24.934	0.035	–0.005	–0.001	0.013
	Scalar		247.699	106	<0.001	0.954	0.051	0.049	67.865	<0.001	–0.009	0.002	0.003
	Factor vriance		255.133	110	<0.001	0.952	0.051	0.063	7.928	0.094	–0.002	0.000	0.014
	Residual*		489.852	124	<0.001	0.845	0.086	0.102	127.070	<0.001	–0.109	0.035	0.054
	Partial residual*	1,2,5,6,7,8	144.056	112	<0.001	0.945	0.054	0.050	15.264	0.018	0.009	–0.003	–0.003
Gender	Configural		45.472	24	0.005	0.987	0.053	0.025	−	−	−	−	−
	Metric		46.633	30	0.027	0.990	0.043	0.033	27.551	0.839	0.002	–0.010	0.008
	Scalar		64.176	36	0.003	0.984	0.049	0.040	230.732	<0.001	–0.006	0.006	0.008
	Factor variance		88.158	37	<0.001	0.970	0.065	0.136	162.348	<0.001	–0.014	0.017	0.095
	Residual*		131.510	43	<0.001	0.941	0.085	0.073	45.674	<0.001	–0.042	0.036	0.032
	Partial residual*	2,7	66.057	41	0.013	0.986	0.042	0.039	15.748	0.008	–0.010	0.010	0.009
Age	Configural		63.391	36	0.003	0.986	0.057	0.027	−	−	−	−	−
	Metric		75.737	48	0.007	0.985	0.051	0.049	134.609	0.336	–0.001	–0.006	0.022
	Scalar		95.195	60	0.003	0.983	0.049	0.052	198.170	0.071	–0.002	–0.002	0.003
	Factor variance		95.908	62	0.004	0.983	0.048	0.064	18.565	0.395	0.000	–0.001	0.011
	Residual*		101.703	74	0.018	0.985	0.042	0.053	11.880	0.616	0.002	–0.007	0.001
Education	Configural		66.657	36	0.001	0.984	0.060	0.026	−	−	−	−	−
	Metric		83.912	48	0.001	0.980	0.059	0.050	183.622	0.105	–0.005	–0.001	0.024
	Scalar		110.261	60	<0.001	0.974	0.060	0.053	301.806	0.003	–0.005	0.000	0.003
	Factor variance		120.791	62	<0.001	0.969	0.064	0.100	75.149	0.023	–0.005	0.005	0.046
	Residual*		123.384	74	<0.001	0.971	0.057	0.065	17.481	0.231	–0.003	–0.003	0.011
T2D status	Configural		40.073	24	0.021	0.991	0.046	0.023	−	−	−	−	−
	Metric		42.627	30	0.063	0.992	0.038	0.029	42.037	0.649	0.001	–0.008	0.006
	Scalar		50.677	36	0.053	0.992	0.036	0.030	76.128	0.268	0.000	–0.002	0.001
	Factor variance		50.969	37	0.063	0.992	0.035	0.038	0.693	0.405	0.000	–0.001	0.008
	Residual*		66.057	43	0.013	0.986	0.042	0.039	14.072	0.050	–0.006	0.007	0.008
Time	Configural		84.234	36	<0.001	0.988	0.051	0.022	−	−	−	−	−
	Metric		94.655	48	<0.001	0.988	0.044	0.032	11.560	0.482	0.000	–0.007	0.010
	Scalar		135.387	60	<0.001	0.982	0.048	0.038	54.319	<0.001	–0.006	0.003	0.005
	Factor variance		149.413	62	<0.001	0.979	0.051	0.074	9.799	0.007	–0.003	0.004	0.036
	Residual*		210.355	74	<0.001	0.964	0.061	0.058	61.287	<0.001	–0.018	0.013	0.021
	Partial residual*	4,5	66.057	70	0.013	0.986	0.042	0.039	14.077	0.170	–0.001	–0.002	0.003

df, degrees of freedom; Δχ2, change in χ2; CFI, comparative fit index; RMSEA, root-mean-square error of approximation; SRMR, standardized root mean square residual: ΔCFI, change in CFI; ΔRMSEA, change in RMSEA; ΔSRMR, change in SRMR; T2D, type two diabetes. *Compared to the model with constrained loadings and intercepts (i.e., the scalar invariant model).

#### GAD-7

Applying CFA resulted in excellent model fit for all different subgroups using the one-factor model with correlated residuals (see Table 3). Scalar invariance could be established across all different subgroups (see Table 4). Invariance of latent factor variance could be established for all subgroups, except for gender. Full or partial (for gender and measurement occasions) residual invariance could be established across all subgroups.

### Group Differences

For both scales, group differences in symptom severity calculated from unit-weighted scores versus latent mean scores show similar results, although the effect size based on latent mean scores was systematically higher (see Table 5). This difference was more pronounced for the GAD-7. Symptom severity scores were higher among women and groups with lower educational attainment. There was no difference between people with or at risk of T2D. Age groups did not differ, except for higher PHQ-9 scores among the eldest cohort. Severity scores decreased over different measurement occasions.

**TABLE 5:** Descriptive statistics of the PHQ-9 and GAD-7 per category and comparison of effect sizes between unit-weighted and latent means.

			**Unit-weighted scores**		**Unit-weighted score differences^a^**		**Latent mean differences^b^**	
**Category**	**Subgroup**	***N***	**Mean**	***SD***	**SES**	***P*-value**	**SES**	***P*-value**
**PHQ-9**								
Total		1207	3.71	3.72				
Gender	Female	553	4.66	3.93				
	Male	654	2.91	3.34	−0.417	<0.001	−0.429	<0.001
Age	30–40	326	3.24	3.27				
	41–50	493	3.66	3.78	0.076	0.203	0.094	0.228
	51–60	388	4.16	3.97	0.152	0.016	0.194	0.001
Education	Primary	305	4.83	4.34				
	Secondary	619	3.72	3.67	−0.195	0.001	−0.259	<0.001
	Higher	283	2.45	2.56	−0.446	<0.001	−0.602	<0.001
Status	At risk	1006	3.77	3.77				
	T2D	201	3.4	3.47	−0.046	0.477	−0.085	0.508
Time	T0	1006	3.77	3.77				
	T1	982	3.09	3.49	−0.168	<0.001	−0.151	<0.001
	T2	963	2.68	3.19	−0.255	<0.001	−0.262	<0.001
**GAD-7**								
Total		1205	3.16	3.33				
Gender	Female	554	3.9	3.72				
	Male	651	2.52	2.8	−0.299	<0.001	−0.603	<0.001
Age	30–40	324	3.09	3.19				
	41–50	495	3.09	3.26	−0.039	0.471	0.042	0.583
	51–60	386	3.3	3.53	0.002	0.973	0.130	0.101
Education	Primary	305	3.91	3.81				
	Secondary	617	3.1	3.2	−0.144	0.007	−0.292	<0.001
	Higher	283	2.47	2.86	−0.328	<0.001	−0.541	<0.001
Status	At risk	1003	3.21	3.37				
	T2D	202	2.88	3.11	−0.065	0.265	−0.138	0.181
Time	T0	1003	3.21	3.37				
	T1	981	2.62	2.94	−0.132	<0.001	−0.180	<0.001
	T2	963	2.42	2.81	−0.172	<0.001	−0.256	<0.001

*^a^Based on robust regression with MM-estimation.*

*^*b*^Based on multigroup structural equation modeling using a second order model for the PHQ-9 to allow for comparison of the general factor latent mean. SD, standard deviation; SES, standardized effect size; T2D, type two diabetes. Interpretation of the SES corresponds to Cohen’s d (conventionally: d = 0.20, 0.50, and 0.80 for small, medium, and large effects, respectively). Empty rows indicate the reference category*.

## Discussion

This is the first study assessing the factorial structure, unit-weighted score reliability, and invariance of the PHQ-9 and the GAD-7 in population in South Asia. For both scales, our findings support the presence of a dominant general factor, but also suggest a somatic and a cognitive/affective subcomponent. This was less explicit for the GAD-7. However, this multidimensionality was not sufficient to justify the scoring of subscales. Our findings only supported the use of unit-weighted scores of the full scales. Scales also showed full scalar invariance and partial to full residual invariance across gender, age, education, measurement occasions and T2D status.

For the PHQ-9, only a 2-factor model showed an acceptable fit in our population in support of a somatic and cognitive/affective subcomponent. While initially, previous research proposed a one-factor model for the PHQ-9, this has been attributed to practical reasons, rather than being supported by a conceptual model (Lamela et al., 2020). More recently, and in line with our findings, studies have provided empirical support for a 2-factor model (Lamela et al., 2020) with a somatic and a cognitive/affective factor. This model corresponds with the occurrence of two clinically distinct profiles of symptoms: one with a combination of high levels of cognitive/affective symptoms and low levels of somatic symptoms, and one with a combination of high levels of both subtypes of symptoms and has been supported by neurobiological findings (Lamela et al., 2020). Distinction of these subtypes has been shown to have clinical relevance in terms of prognosis and treatment (Lamela et al., 2020).

For the GAD-7, models with the somatic items as a different group or with their residuals correlated showed an adequate fit, while a unidimensional model did not. This provides empirical support for an overarching construct reflecting symptom severity, but also supports the eventual distinction of a somatic and a cognitive/affective subcomponent. Based on several samples of American psychiatric patients, researchers have argued for a one-factor model with the same correlated residuals, reflecting a method effect (referring to the nature of the question) rather than a somatic subtype (Kertz et al., 2012; Rutter and Brown, 2017; Johnson et al., 2019). Others have argued for a two-factor model, with the same items as one factor (Beard and Björgvinsson, 2014; Moreno et al., 2019). In a sample of Japanese psychiatric patients, a higher order 2-factor model was proposed (Doi et al., 2018a). Model selection in those studies was typically based on their goodness of fit. We argue that beyond these statistical criteria, conclusions should be informed by evidence from other domains. The existence of a worry subtype and a somatic subtype has been suggested based on pathophysiologic findings and clinical manifestations (Portman et al., 2011), however, more evidence is needed regarding their clinical relevance.

Factor loadings of the GAD-7 indicators ranged from acceptable to high, except for item 6. Lower factor loadings of this item have been reported in several other studies (Zhong et al., 2015; Rutter and Brown, 2017; Johnson et al., 2019) albeit not as low as in our study. Despite this low factor loading, reliability indices did not change substantially when the item was excluded. This finding in combination with the scale’s established validation in a variety of settings supports the use of the scale including item 6. However, we recommend further investigation of the item’s wordings and understanding when being used in similar settings.

Our findings indicate that for both scales, although they are not fully unidimensional, the general factor is the major determinant of the variance underlying the unit-weighted total scale scores. This supports unit-weighted scores of the full scales as being a reliable reflection of depression or anxiety symptom severity. However, the Haberman procedure showed that reporting unit-weighted subscale scores does not have an added value as they are not more precise than the total scores in estimating the corresponding subdimension, except for the cognitive dimension of the PHQ-9. In addition, low OmegaHS indices suggested that unit-weighted subscale scores do not provide reliable measures of constructs independently of the general construct. In other words, the subscale scores were redundant with the total score. This is in contrast with recommendations made in recent studies (Doi et al., 2018a,b). Our study expands on their findings which were based on the goodness of fit of a confirmatory factor model; an approach that has been argued insufficient for this purpose (Reise et al., 2013).

Scalar invariance was established for all studied subgroups, which suggests a similar response and interpretation of scale items across these groups (Vandenberg and Lance, 2000). Scalar invariance also justifies comparison using SEM. The use of unit-weighted scores requires invariant indicator reliability that implies invariant residuals after having established invariance of latent factor variances (Vandenberg and Lance, 2000). The latter could not be established for education (PHQ-9 only) and gender (GAD-7 only) which does not rule out indicator reliability, however, precludes the interpretation of residual invariance as indicator reliability. Full or partial residual invariance was found for the remaining subgroups. The effect of residual invariance being partial rather than full has been poorly studied and currently, no agreement exists on the required number of items to obtain an acceptable level of invariance (Putnick and Bornstein, 2016). Some experts argue that two items is sufficient (Putnick and Bornstein, 2016).

The differences in effect size between subgroups turned out to be systematically higher using latent means compared to unit-weighted scores. This is expected since random error is ignored when comparing latent means. However, the difference in effect size was much smaller for the PHQ-9. This can be explained by the use of a second-order model for this scale representing the common effect of the subfactors. Unit-weighted scores and the unidimensional model for the GAD-7 did not distinguish between general and subfactor effects, resulting in relatively higher effect sizes. We conclude that our findings support the use of unit-weighted scores to detect differences of a reasonable effect size across different subgroups. However, we do recommend the use of SEM whenever possible and certainly if a precise estimate is at stake.

Our findings regarding invariance of the PHQ-9 are in line with previous studies conducted in western settings assessing residual invariance for gender and age (Lamela et al., 2020). Those studies also showed residual invariance for education. Full or partial (after freeing 1 item) residual invariance over different measurement occasions was found among primary care patients (González-Blanch et al., 2018) and COPD patients (Schuler et al., 2018). However, time intervals were smaller (3 months) in those studies. Our findings on invariance of the GAD-7 are in line with previous studies conducted in western settings reporting scalar invariance across gender and age (Hinz et al., 2017; Rutter and Brown, 2017); residual invariance was not tested in these studies. A study using a digitalized version of the GAD-7 found residual invariance across gender, age, education and different measurement occasions (Moreno et al., 2019). For both scales, our study is the first to establish invariance between people at risk of T2D and with T2D.

Higher severity scores of depression and anxiety in women and people with lower education are in line with national and global trends (Bjelland et al., 2008; Grover et al., 2010; Hinz et al., 2017; Sagar et al., 2020). Prevalence of both disorders followed the same trends. Higher severity scores of depression in the older subgroup and the status quo of anxiety severity across age groups were compatible with national trends (Sagar et al., 2020).

Our findings are in support of the use of both scales to measure symptom severity among individuals. However, health workers need to take into account that part of the scores does not reflect symptom severity, but random error, and this part differs across individuals. Indicator reliability across certain categories (e.g., gender and education) may also slightly vary since full residual invariance could not be established. Moreover, the lack of full residual invariance over time suggests that scores’ reliability may vary over time (i.e., years) which should be taken into account when using the scales for patient follow-up. We conclude that, while scores of an individual may give an idea of symptom severity, further examination is essential for an accurate diagnosis.

### Strengths and Limitations

A major strength of our study was the use of a large sample size which was generally representative of Kerala’s general population in terms of age structure, education, occupation, marital status, and household size at the time of study enrollment (Office of the Registrar General and Census Commissioner of India, 2011). However, the gender ratio was lower than the state’s average and the study population was restricted to people aged 30–60 years, with no history of chronic conditions yet at risk of diabetes. Generalizing of our findings to other states of India also warrants caution since Kerala’s literacy rate and health indicators are better than most Indian states. However, since the scales showed invariance for age, gender, education and status of diabetes, we assume that differences in these characteristics may not have a substantial influence. We conducted an extensive assessment of reliability based on bifactor modeling and compared differences between unit-weighted scores and latent means. To our knowledge, these techniques have not been applied previously to these scales. A weakness of the study is that we did not examine criterion validity nor did we evaluate invariance for ethnicity and socioeconomic status. Another limitation was the use of a maximum likelihood estimator for the parameter estimation of the factor analyses, which likely contributed to a negative bias in parameter estimates because of the non-normal data (Li, 2016). Weighted least square estimation would normally be preferable as it has been shown more accurate if the normality assumption is violated (Li, 2016). However, the use of tetrachoric or polychoric correlations has been shown to overestimate reliability (Revelle and Condon, 2018). A model-based reliability index for categorical data has been proposed, but evidence about its performance is lacking (Green and Yang, 2009). Since assessment of reliability was the primary objective of this study, we therefore opted for a maximum likelihood estimator. As expected, sensitivity analysis with diagonally weighted least squares resulted in higher factor loadings and improved model fit. Despite this, the difference was not large enough to alter our conclusions. Using the formula proposed by Green and Yang resulted in similar estimates of reliability: 0.73 for PHQ-9 and 0.79 for GAD-7. Finally, it would be interesting for future studies to study invariance of response patterns across global regions.

In conclusion, our findings support the existence of a somatic and a cognitive subtype of depression and to a lesser extent for anxiety in a rural Indian population. Unit-weighted scores of the full scales can be used at individual and population level, reflecting a single construct of symptom severity. However, one needs to take into account that part of the score corresponds to error. Scoring of the subscales is redundant. Both scales showed to be stable across demographic subgroups and over time, which supports their use and allows for meaningful comparison across tested subgroups in the Indian context. For both scales, psychometric properties are comparable to studies in western settings.

## Data Availability Statement

The raw data supporting the conclusions of this article will be made available by the authors, without undue reservation.

## Ethics Statement

The studies involving human participants were reviewed and approved by the Institutional Ethics Committee of the Sree Chitra Tirunal Institute for Medical Sciences and Technology, Trivandrum, Kerala, and by the Human Research Ethics Committees of Monash University, Australia and the University of Melbourne, Australia. The study was also approved by the Health Ministry Screening Committee of the Government of India. The patients/participants provided their written informed consent to participate in this study.

## Author Contributions

PA, TS, BO, JD, and EW played a major role in the conception of the study. PA, TS, KT, BO, and EW contributed to the design of the study. JD drafted the manuscript and analyzed and interpreted the data. PA, LJ, AD, TS, TH, KT, BO, and EW critically revised the manuscript for important intellectual content, and read and approved the final version. All authors contributed to the article and approved the submitted version.

## Conflict of Interest

The authors declare that the research was conducted in the absence of any commercial or financial relationships that could be construed as a potential conflict of interest.

## References

[B1] Beard C. Björgvinsson T. (2014). Beyond generalized anxiety disorder: psychometric properties of the GAD-7 in a heterogeneous psychiatric sample. *J. Anxiety Disord.* 28 547–552. 10.1016/j.janxdis.2014.06.002 24983795

[B2] Bjelland I. Krokstad S. Mykletun A. Dahl A. A. Tell G. S. Tambs K. (2008). Does a higher educational level protect against anxiety and depression? The HUNT study. *Soc. Sci. Med.* 66 1334–1345. 10.1016/j.socscimed.2007.12.019 18234406

[B3] Brosseau-Liard P. E. Savalei V. Li L. (2012). An investigation of the sample performance of two nonnormality corrections for RMSEA. *Multivariate Behav. Res.* 47 904–930. 10.1080/00273171.2012.715252 26735008

[B4] Dere J. Watters C. A. Yu S. C. M. Michael Bagby R. Ryder A. G. Harkness K. L. (2015). Cross-cultural examination of measurement invariance of the beck depression inventory-II. *Psychol. Assess.* 27 68–81. 10.1037/pas0000026 25314096

[B5] Doi S. Ito M. Takebayashi Y. Muramatsu K. Horikoshi M. (2018a). Factorial validity and invariance of the 7-item generalized anxiety disorder scale (GAD-7) among populations with and without self-reported psychiatric diagnostic status. *Front. Psychol.* 9:1741. 10.3389/fpsyg.2018.01741 30283386 PMC6157449

[B6] Doi S. Ito M. Takebayashi Y. Muramatsu K. Horikoshi M. (2018b). Factorial validity and invariance of the Patient Health Questionnaire (PHQ)-9 among clinical and non-clinical populations. *PLoS One* 13:e0199235. 10.1371/journal.pone.0199235 30024876 PMC6053131

[B7] Duivis H. E. Vogelzangs N. Kupper N. De Jonge P. Penninx B. W. J. H. (2013). Differential association of somatic and cognitive symptoms of depression and anxiety with inflammation: findings from the netherlands study of depression and anxiety (NESDA). *Psychoneuroendocrinology* 38 1573–1585. 10.1016/j.psyneuen.2013.01.002 23399050

[B8] Eid M. Geiser C. Koch T. Heene M. Eid M. Geiser C. (2016). Psychological methods alternatives anomalous results in G -factor models: explanations and alternatives. *Psychol. Methods* 22 541–562. 27732052 10.1037/met0000083

[B9] Estabrook R. Sadler M. E. McGue M. (2015). Differential item functioning in the cambridge mental disorders in the elderly (CAMDEX) depression scale across middle age and late life. *Psychol. Assess.* 27 1219–1233. 10.1037/pas0000114 25938337 PMC4633409

[B10] González-Blanch C. Medrano L. A. Muñoz-Navarro R. Ruíz-Rodríguez P. Moriana J. A. Limonero J. T. (2018). Factor structure and measurement invariance across various demographic groups and over time for the PHQ-9 in primary care patients in Spain. *PLoS One* 13:e0193356. 10.1371/journal.pone.0193356 29474410 PMC5825085

[B11] Green S. B. Yang Y. (2009). Reliability of summed item scores using structural equation modeling: an alternative to coefficient alpha. *Psychometrika* 74 155–167. 10.1007/s11336-008-9099-3

[B12] Grover S. Dutt A. Avasthi A. (2010). An overview of Indian research in depression. *Indian J. Psychiatry* 52:178. 10.4103/0019-5545.69231 21836676 PMC3146226

[B13] Haberman S. J. (2008). When can subscores have value? *J. Educ. Behav. Stat.* 33 204–229. 10.3102/1076998607302636

[B14] Hinz A. Klein A. M. Brähler E. Glaesmer H. Luck T. Riedel-Heller S. G. (2017). Psychometric evaluation of the generalized anxiety disorder screener GAD-7, based on a large german general population sample. *J. Affect. Disord.* 210 338–344. 10.1016/j.jad.2016.12.012 28088111

[B15] Hu L. Bentler P. M. (1999). Cutoff criteria for fit indexes in covariance structure analysis: conventional criteria versus new alternatives. *Struct. Equ. Model. A Multidiscip. J.* 6 1–55. 10.1080/10705519909540118

[B16] Johnson S. U. Ulvenes P. G. Øktedalen T. Hoffart A. (2019). Psychometric properties of the GAD-7 in a heterogeneous psychiatric sample. *Front. Psychol.* 10:1713. 10.3389/fpsyg.2019.01713 31447721 PMC6691128

[B17] Jorgensen T. D. Pornprasertmanit S. Schoemann A. M. Rosseel Y. (2020). *Semtools: Useful Tools for Structural Equation Modeling. R Package Version 0.5-3.* Available online at: https://cran.r-project.org/package=semTools [Accessed June 1, 2020].

[B18] Kertz S. Bigda-Peyton J. Bjorgvinsson T. (2012). Validity of the generalized anxiety disorder-7 scale in an acute psychiatric sample. *Clin. Psychol. Psychother.* 20 456–464. 10.1002/cpp.1802 22593009

[B19] Kroenke K. Spitzer R. L. Williams J. B. W. (2001). The PHQ-9. *J. Gen. Intern. Med.* 16 606–613. 10.1046/j.1525-1497.2001.016009606.x 11556941 PMC1495268

[B20] Kroenke K. Spitzer R. L. Williams J. B. W. Löwe B. (2010). The patient health questionnaire somatic, anxiety, and depressive symptom scales: a systematic review. gen. hosp. *Psychiatry* 32 345–359. 10.1016/j.genhosppsych.2010.03.006 20633738

[B21] Lamela D. Soreira C. Matos P. Morais A. (2020). Systematic review of the factor structure and measurement invariance of the patient health questionnaire-9 (PHQ-9) and validation of the Portuguese version in community settings. *J. Affect. Disord.* 276 220–233. 10.1016/j.jad.2020.06.066 32697702

[B22] Leong F. T. L. Tak J. (2003). Assessment of depression and anxiety in East Asia. *Psychol. Assess.* 15 290–305. 10.1037/1040-3590.15.3.290 14593829

[B23] Li C. H. (2016). Confirmatory factor analysis with ordinal data: comparing robust maximum likelihood and diagonally weighted least squares. *Behav. Res. Methods* 48 936–949. 10.3758/s13428-015-0619-7 26174714

[B24] Miranda C. A. C. Scoppetta O. (2018). Factorial structure of the Patient Health Questionnaire-9 as a depression screening instrument for university students in Cartagena. *Colombia. Psychiatry Res.* 269 425–429. 10.1016/j.psychres.2018.08.071 30195230

[B25] Moreno E. Muñoz-Navarro R. Medrano L. A. González-Blanch C. Ruiz-Rodríguez P. Limonero J. T. (2019). Factorial invariance of a computerized version of the GAD-7 across various demographic groups and over time in primary care patients. *J. Affect. Disord.* 252 114–121. 10.1016/j.jad.2019.04.032 30981054

[B26] Mughal A. Y. Devadas J. Ardman E. Levis B. Go V. F. Gaynes B. N. (2020). A systematic review of validated screening tools for anxiety disorders and PTSD in low to middle income countries. *BMC Psychiatry* 20:338. 10.1186/s12888-020-02753-3 32605551 PMC7325104

[B27] Office of the Registrar General and Census Commissioner of India (2011). *Census 2011.* Available online at: http://censusindia.gov.in (accessed November 9, 2020)

[B28] Portman M. E. Starcevic V. Beck A. T. (2011). Challenges in assessment and diagnosis of generalized anxiety disorder. *Psychiatr. Ann.* 41 79–85. 10.3928/00485713-20110203-06

[B29] Putnick D. L. Bornstein M. H. (2016). Measurement invariance conventions and reporting: the state of the art and future directions for psychological research. *Dev. Rev.* 41 71–90. 10.1016/j.dr.2016.06.004 27942093 PMC5145197

[B30] Reise S. P. Bonifay W. E. Haviland M. G. (2013). Scoring and modeling psychological measures in the presence of multidimensionality. *J. Pers. Assess.* 95 129–140. 10.1080/00223891.2012.725437 23030794

[B31] Revelle W. Condon D. (2018). Reliability from alpha to omega: a tutorial. *[preprint]* 10.31234/osf.io/2y3w9 31380696

[B32] Rodriguez A. Reise S. P. Haviland M. G. (2016a). Applying bifactor statistical indices in the evaluation of psychological measures. *J. Pers. Assess.* 98 223–237. 10.1080/00223891.2015.1089249 26514921

[B33] Rodriguez A. Reise S. P. Haviland M. G. (2016b). Evaluating bifactor models: calculating and interpreting statistical indices. *Psychol. Methods* 21 137–150. 10.1037/met0000045 26523435

[B34] Rosseel Y. (2012). Lavaan: an R package for structural equation modeling. *J. Stat. Softw* 48 1–36. 10.18637/jss.v048.i02

[B35] Rutter L. A. Brown T. A. (2017). Psychometric Properties of the generalized anxiety disorder scale-7 (GAD-7) in outpatients with anxiety and mood disorders. *J. Psychopathol. Behav. Assess.* 39 140–146. 10.1007/s10862-016-9571-9 28260835 PMC5333929

[B36] Sagar R. Dandona R. Gururaj G. Dhaliwal R. S. Singh A. Ferrari A. (2020). The burden of mental disorders across the states of India: the global burden of disease study 1990–2017. *Lancet Psychiatry* 7 148–161. 10.1016/S2215-0366(19)30475-4 31879245 PMC7029418

[B37] Sathish T. Aziz Z. Absetz P. Thankappan K. R. Tapp R. J. Balachandran S. (2019). Participant recruitment into a community-based diabetes prevention trial in India: learnings from the Kerala diabetes prevention program. *Contemp. Clin. Trials Commun.* 15:100382. 10.1016/j.conctc.2019.100382 31193921 PMC6545388

[B38] Sathish T. Oldenburg B. Tapp R. J. Shaw J. E. Wolfe R. Sajitha B. (2017). Baseline characteristics of participants in the Kerala diabetes prevention program: a cluster randomized controlled trial of lifestyle intervention in Asian Indians. *Diabet. Med.* 34 647–653. 10.1111/dme.13165 27279083 PMC5148720

[B39] Sathish T. Williams E. D. Pasricha N. Absetz P. Lorgelly P. Wolfe R. (2013). Cluster randomised controlled trial of a peer-led lifestyle intervention program: study protocol for the Kerala diabetes prevention program. *BMC Public Health* 13:1035. 10.1186/1471-2458-13-1035 24180316 PMC3937241

[B40] Schuler M. Strohmayer M. Mühlig S. Schwaighofer B. Wittmann M. Faller H. (2018). Assessment of depression before and after inpatient rehabilitation in COPD patients: psychometric properties of the German version of the Patient Health Questionnaire (PHQ-9/PHQ-2). *J. Affect. Disord.* 232 268–275. 10.1016/j.jad.2018.02.037 29499510

[B41] Sinharay S. (2019). Added value of subscores and hypothesis testing. *J. Educ. Behav. Stat.* 44 25–44. 10.3102/1076998618788862

[B42] Spitzer R. L. Kroenke K. Williams J. B. W. Löwe B. (2006). A brief measure for assessing generalized anxiety disorder: the GAD-7. *Arch. Intern. Med.* 166 1092–1097. 10.1001/archinte.166.10.1092 16717171

[B43] Steinmetz H. (2013). Analyzing observed composite differences across groups. *Methodology* 9 1–12. 10.1027/1614-2241/a000049

[B44] Vandenberg R. J. Lance C. E. (2000). A review and synthesis of the measurement invariance literature: suggestions, practices, and recommendations for organizational research. *Organ. Res. Methods* 3 4–70. 10.1177/109442810031002

[B45] Vos T. Barber R. M. Bell B. Bertozzi-Villa A. Biryukov S. Bolliger I. (2015). Global, regional, and national incidence, prevalence, and years lived with disability for 301 acute and chronic diseases and injuries in 188 countries, 1990–2013: a systematic analysis for the global burden of disease study 2013. *Lancet* 386 743–800. 10.1016/S0140-6736(15)60692-4 26063472 PMC4561509

[B46] World Health Organization. (2008). *The Global Burden of Disease:2004 Update.* Available online at: https://www.who.int/healthinfo/global_burden_disease/GBD_report_2004update_full.pdf (accessed May 23, 2020)

[B47] Zhong C. Gelaye Q.-Y. Zaslavsky B. M. Fann A. M. Rondon J. R. (2015). Diagnostic validity of the generalized anxiety disorder-7 (GAD-7) among pregnant women. *PLoS One* 10:125096. 10.1371/journal.pone.0125096 25915929 PMC4411061

[B48] Zinbarg R. E. Revelle W. Yovel I. Li W. (2005). Cronbach’s, α revelle’s β and McDonald’s ω H: their relations with each other and two alternative conceptualizations of reliability. *Psychometrika* 70 123–133. 10.1007/s11336-003-0974-7

[B49] Zinbarg R. E. Revelle W. Yovel I. (2007). Estimating ωh for structures containing two group factors: Perils and prospects. *Appl. Psychol. Meas.* 31 135–157. 10.1177/0146621606291558

